# A practical 10-step recipe for conducting radiomic studies

**DOI:** 10.1186/s41747-025-00666-y

**Published:** 2026-03-02

**Authors:** Blanca Rodriguez-Gonzalez, Alberto Martinez-Caballero, Javier Soto-Perez-Olivares, Jaime Moujir-Lopez, Javier Blazquez-Sanchez, Borja Rodriguez-Vila, Angel Torrado-Carvajal

**Affiliations:** 1 Medical Image Analysis and Biometry Laboratory, Universidad Rey Juan Carlos, Madrid, Spain; 2 Department of Radiology, Hospital Universitario Ramón y Cajal, IRYCIS, Madrid, Spain

**Keywords:** Artificial intelligence, Diagnostic imaging, Machine learning, Radiomics, Precision medicine

## Abstract

**Abstract:**

Radiomics is a growing field in medical imaging that transforms images into high-dimensional quantitative data, offering insights into disease diagnosis, prognosis, and treatment planning. Using advanced computational techniques, radiomics uncovers patterns invisible to the human eye, playing a key role in precision medicine. However, the adoption of radiomics faces several barriers, including a lack of standardization, reproducibility challenges, and difficulties in clinical implementation. To address these challenges, a practical 10-step recipe is proposed to guide researchers in conducting effective radiomic studies: (1) identify a genuine clinical need and application; (2) establish a comprehensive database; (3) implement robust quality assurance and preprocessing; (4) ensure accurate image segmentation; (5) extract quantitative imaging features; (6) prioritize feature selection and dimension reduction; (7) consider integration of clinical and multi-omics data; (8) construct predictive models with machine learning techniques; (9) evaluate model performance using appropriate metrics; (10) translate models into clinical practice and workflow integration. This recipe emphasizes research rationale and methodologies, ensuring that the studies are aligned with real clinical needs, employing advanced techniques, and promoting reproducibility. By addressing these challenges through a structured approach, radiomics can transition from a research discipline to a clinical tool, contributing to more personalized and effective patient care.

**Relevance statement:**

A structured 10-step framework is proposed to guide radiomic research, addressing key challenges in standardization and implementation. This practical guide supports any professional aiming to start in radiomics or adopt best practices, promoting reproducibility and clinical relevance in precision imaging workflows.

**Key Points:**

- Radiomics extracts quantitative data from medical images for improved diagnosis and treatment.
- Reproducibility, standardization issues, and clinical implementation barriers are among the main challenges of the technique.
- Data quality, feature selection, and machine learning are key to meaningful analysis.
- A structured 10-step guide for conducting reliable radiomic studies is proposed, taking a step toward a standardized workflow.

**Graphical Abstract:**

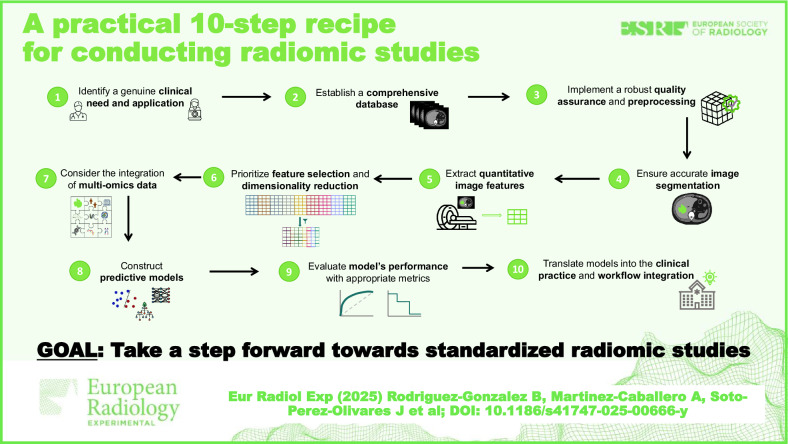

## Introduction

Medical imaging has become an indispensable tool for the diagnosis, treatment, and monitoring of various health conditions, providing healthcare professionals with a non-invasive tool to visualize the internal structures of the human body. Traditionally, the interpretation of these images relies on the expertise of radiologists, who identify structural and textural patterns that aid in the precise characterization of the images. Although trained radiologists excel at this task, this process is highly subjective and heavily reliant on their experience, highlighting the need for a more objective analysis.

Although generally understood as visual data, medical images are much more than simple pictures; they contain significant information in the form of three-dimensional matrices, making them suitable for advanced quantitative analysis. The use of sophisticated computational and mathematical techniques on medical images can uncover information that is invisible to the naked human eye, discovering new disease-specific patterns by using high-dimensional image-derived macroscopic features. Under this hypothesis, the term radiomics has been introduced [1, 2].

Radiomics, derived from the -omics sciences, focuses on extracting a vast number of characteristics from a specific volume of interest, transforming hidden visual information into quantitative data. In general, -omics sciences, including genomics, proteomics, and metabolomics, among others, focus on large-scale data analysis to understand the molecular and cellular mechanisms of biological systems. Similarly, radiomics applies these techniques to imaging, characterizing a volume of interest through quantitative measures. Subsequently, these data are analyzed using advanced algorithms and statistical tools to discover associations with clinical outcomes and other medical factors, leading to more personalized patient care [3].

Despite its potential, radiomics faces several challenges, mainly due to a lack of standardization in image acquisition, data analysis, and feature management. Variations in imaging protocols, differences in equipment, and inconsistencies in software tools can cause significant discrepancies in results, making it difficult to generalize the findings between different studies [4]. Moreover, the absence of standardized methodologies compromises the reproducibility of radiomic studies, creating a significant barrier to their widespread clinical adoption [5, 6].

**Fig. 1:**
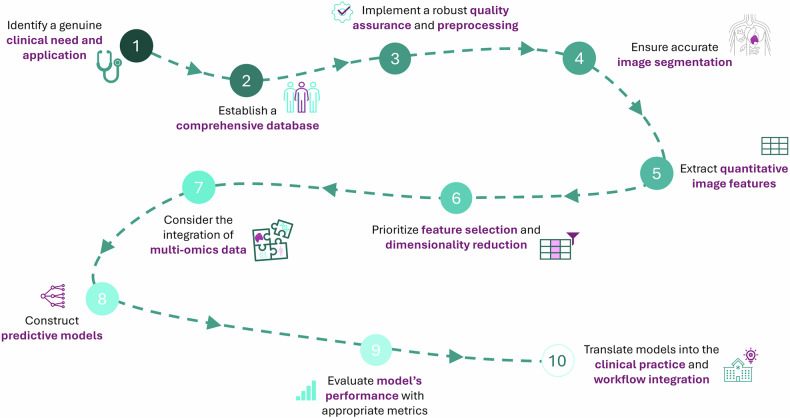
Practical recipe for radiomic studies. Ten steps to follow when performing radiomic studies, from clinical need identification to the integration of the final model into practice

Here, we provide a practical step-by-step guide on how to conduct a radiomic study, outlining the key stages involved in the process. This workflow, summarized in Fig. 1, is designed to offer a clear and structured approach, ensuring reproducibility and robustness in radiomic research. The steps presented are based on our experience and are supported by established methodologies in the literature.

## Step 1: Identify a genuine clinical need and application

Before beginning to design a radiomic study, it is essential to identify a real need. Radiomics should not be applied merely for the sake of applying it; instead, a clinically relevant problem must be encountered.

This technique has the potential to address a wide variety of medical issues, including the screening and detection of certain diseases [7, 8], tumor staging and characterization [9], prediction of the treatment response [10], prognosis of the disease [11], and understanding the pathological mechanisms reflected in images that may relate to biological markers [12].

**Fig. 2:**
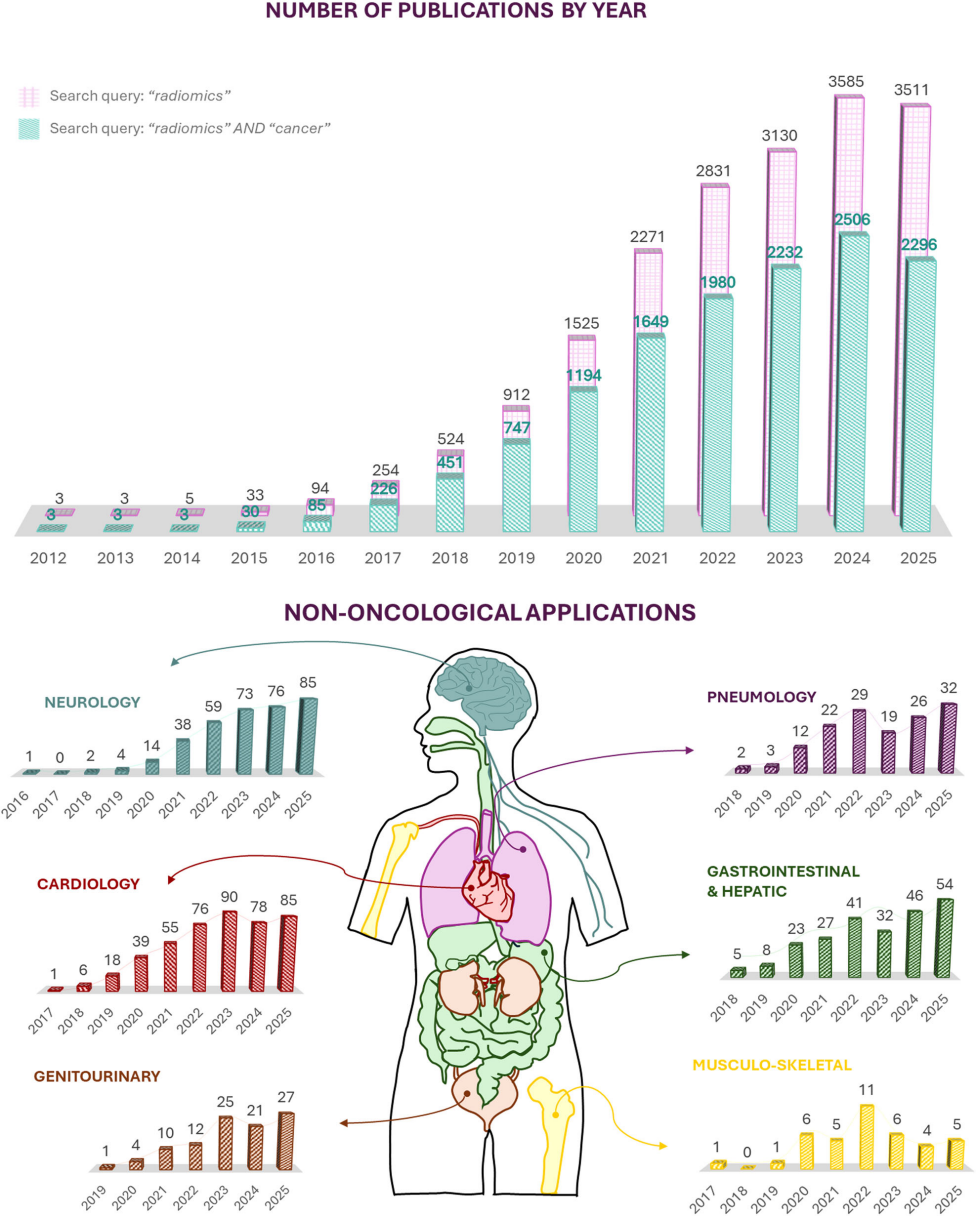
The evolution of radiomics-related scientific output indexed in PubMed from 2012 through 2025 (data accessed on October 21, 2025). The upper panel displays the annual number of publications retrieved using the queries “radiomics” (pink) and “radiomics AND cancer” (teal). A marked increase in publication volume is observed over time, with a consistent predominance of oncology-focused studies. The lower panel illustrates non-oncological applications of radiomics across various medical specialties. These data were obtained using the query “radiomics” in combination with domain-specific keywords representing each specialty

Radiomic analysis was born and has been applied mainly in the field of oncology, where the study of tumors, lymphatic nodes, and other affected areas can lead to a more personalized treatment of patients. However, as a rapidly evolving technique, radiomics can be (and is) applied to other clinical areas, including neurology (*e.g*., prediction of disability in multiple sclerosis [13]) and cardiology (*e.g*., prediction of sudden cardiac death [14]), among others. This tendency is illustrated in Fig. 2.

Its versatility allows radiomics to be a valuable tool in numerous domains, enhancing diagnostic accuracy, treatment planning, personalized medicine, and overall patient management. However, defining a research hypothesis before starting with algorithmics is essential to conduct relevant studies. Without a focused hypothesis, studies risk becoming exploratory exercises with limited translational value.

In this context, it is crucial to resist the temptation of applying the latest algorithmic trends just because they are cutting-edge. While technological innovation is a driving force in radiomics, its application must be guided by clinical relevance rather than methodological novelty. The uncritical use of complex models without a well-defined clinical question can lead to overfitting and reduced interpretability.

To ensure that radiomic studies remain clinically grounded, the design process should be done in interdisciplinary collaboration. Clinicians, imaging experts, and data scientists must work together from the outset to define meaningful objectives. Structured frameworks such as Population, Intervention, Comparison, Outcome (PICO) offer a valuable scaffold for this process, helping to translate clinical needs into actionable research questions [15]. By aligning methodological choices with clinical priorities, researchers can ensure that radiomics serves its intended purpose: solving real problems and improving patient care.

## Step 2: Establish a comprehensive database

Data forms the foundation of radiomics. To create a suitable dataset, medical images and labels should be included. The images constitute the input from which the radiomic features are extracted. These can come from any medical imaging technique, with magnetic resonance imaging (MRI), computed tomography (CT), and positron emission tomography (PET) scans being the most widely used modalities [16]. Sometimes, images from different modalities may be used. On the other hand, labels completely depend on the specific hypothesis being investigated, varying their nature from Boolean nature in classification problems (*i.e*., identifying node affection in lung cancer) to continuous numbers in regression tasks (*i.e*., predicting patient survival time or progression-free survival). It is essential to ensure that these labels are derived from a clearly defined and temporally appropriate reference standard, such as histopathology, molecular profiling, or clinical follow-up. The timing of the reference standard relative to image acquisition is critical to ensure label accuracy and avoid introducing bias or misclassification. An error in label definition or poor-quality labels can compromise the validity of the whole study.

The design of the dataset can follow either a retrospective or a prospective scheme. A retrospective study involves using data collected for other purposes, like daily clinical routine or other research studies. Although cost-effective, this approach usually comes with incomplete data and/or big variability in imaging protocols, which introduces significant bias. On the contrary, under a prospective design, data will be collected specifically for the study, allowing us to create custom imaging protocols and patient selection. Generally, prospective designs are preferred for their ability to ensure complete, homogeneous, and high-quality images; however, due to resource and time constraints, retrospective schemes are usually the ones found.

Data at different temporal points might also be retrieved to observe changes in imaging features over time, which can be particularly valuable in understanding disease progression, treatment response, or recurrence rates. For instance, by analyzing pre-treatment and post-treatment images, radiomic features can help assess the efficacy of a therapeutic intervention or monitor tumor evolution. This approach is sometimes referred to as delta radiomics [17].

Sometimes, the use of public databases can help researchers in their radiomics research, since they provide access to large, usually standardized and diverse datasets. These databases support reproducibility and transparency by providing data for validation and comparison. Although they may not always align perfectly with the specific research question at hand, they can still serve as valuable proof-of-concept tools, allowing researchers to refine their studies, define further milestones, and create better protocols for future, more targeted studies.

Additionally, incorporating data from multiple centers can significantly enhance the generalizability and robustness of radiomic models. Multicenter datasets offer a broader representation of patient populations, imaging equipment, and clinical practices. However, this approach must be handled with care. Inconsistent imaging protocols across centers can introduce substantial bias and variability in extracted features. If not properly accounted for, these discrepancies may mitigate true biological signals and compromise model performance.

To mitigate these risks, harmonization strategies should be employed, such as Combatting Batch Effect‒ComBat, which adjusts for batch effects while preserving biological variability [18]. When integrating multicenter data, it is also advisable to document acquisition parameters meticulously and, when possible, stratify analyses by center or include center-specific covariates in modeling.

## Step 3: Implement robust quality assurance and preprocessing

Radiomics relies on numbers, and numbers are directly derived from images. High-quality, homogeneous medical images are the central piece of our research, directly influencing the accuracy of posterior analyses. When garbage comes in, garbage will go out, *i.e*., poor-quality images will inevitably lead to erroneous results and conclusions, no matter how advanced the analytical techniques used are.

To avoid this, quality assurance measures must be taken. These include ensuring consistency in imaging protocols, guaranteeing homogeneous intensity ranges between images, and isotropic voxel sizes. Sometimes, visual inspection is additionally required to further ensure data integrity. Questions like “Is the image resolution adequate? Are there any visible artifacts or noise in the images? Is there consistency in image orientation and positioning across the dataset? Or is there sufficient coverage of the target area?” should be considered. Ignoring these questions may lead to non-comparable features, undermining the reliability of the entire analysis.

Prospective databases can significantly enhance data quality since, by following consistent imaging practices, data variability is minimized and quality is maintained across the dataset. In contrast, retrospective databases often require an additional preprocessing step.

Preprocessing techniques play a crucial role in preparing images for analysis, helping to standardize and improve their quality when standards are not fulfilled. Preprocessing steps may include:

- image resampling to a uniform voxel size to ensure that the features extracted from different images can be compared. This size homogeneity can be achieved by using interpolation algorithms;
- gray-level discretization to a standard intensity range to warrant the robustness of texture analysis;
- denoising techniques, such as Gaussian filtering or wavelet transforms, are also employed to remove unwanted noise while preserving essential details.

Programming environments like Python or MATLAB contain specialized functions and packages to carry out these operations, including libraries like Python’s SimpleITK [19] or scikit-image [20]. Other engines like 3DSlicer [21], or ANTs [22] offer an interactive framework for this task. The one to use will depend completely on the researcher’s expertise.

It is important to highlight that preprocessing steps must be applied with caution. Overprocessing can degrade image quality, leading to the loss of information, distorting features and adversely affecting the outcomes of the analysis. Balance needs to be found.

## Step 4: Ensure accurate image segmentation

Although an overall image is required, radiomic analysis is usually based on the detailed study of a concrete area related to our specific hypothesis. Image segmentation becomes, therefore, essential for radiomic analysis.

Technically, image segmentation refers to the task of dividing an image into different regions based on certain criteria of similarity (for example, pixels’ intensity level). These regions, often referred to as regions of interest (ROIs), should not overlap and must define significant structures with, in the case of medical image, anatomical or functional sense [23].

While originally performed manually, image segmentation methods have significantly evolved since the late 1970s, with early digital image processing introducing semi-automatic techniques, until today, when cutting-edge technologies like artificial intelligence (AI) have made fully automatic segmentation methods a reality. All these approaches might be applied when delineating the area (or areas) of interest in a radiomic study, each with its own strengths and limitations.

Manual segmentation, performed by a trained expert or radiologist who manually outlines the ROIs using medical imaging software, offers high precision and the ability to adapt to specific cases, capturing specific details. However, this approach is labor- and cost-intensive and subject to variability between different raters, making it impractical (and sometimes impossible) for large datasets.

To address these challenges, semi-automatic segmentation methods combine user input with automated algorithms. Semi-automatic algorithms often begin with seeds defined by an expert, indicating relevant points of the structure that guide the algorithm in identifying the complete target regions in the image by propagating these initial inputs. Examples of such algorithms include region growing, expectation-maximization and/or watershed [24]. After the process, inaccuracies of the methods can be additionally corrected by the expert, ensuring an appropriate segmentation. This hybrid approach speeds up the process while still allowing for expert refinement, reducing the overall manual workload but still dependent on the rater’s experience and approach.

The rise of AI, particularly in the realm of deep learning (DL) techniques, has led to the development of fully automated segmentation methods as a powerful alternative. DL-based approaches, especially convolutional neural networks, have demonstrated success in segmenting complex anatomical structures across various imaging modalities such as CT, MRI, and PET. These models are capable of automatically learning hierarchical features, which are essential for differentiating between distinct regions. Architectures based on U-Nets and their more advanced variants, including the integration of attention blocks, are among the most commonly used for achieving precise segmentation [25, 26]. This fully automated approach significantly reduces the time and cost required for segmentation in radiomic studies. However, blind trust in automatic segmentation without proper validation can be misleading. Automated methods, while efficient, may produce inconsistent or inaccurate results if not rigorously evaluated.

Depending on the specifics of our study, we will select the most appropriate method. In some cases, when working with a small cohort of cases from a single center, manual segmentation may be preferred since it will allow for greater accuracy and the incorporation of expert knowledge. On the other hand, when working with a large, multicenter dataset, automatic segmentation might be chosen to reduce the time required for processing, minimize human error, and ensure consistency across diverse datasets.

**Fig. 3:**
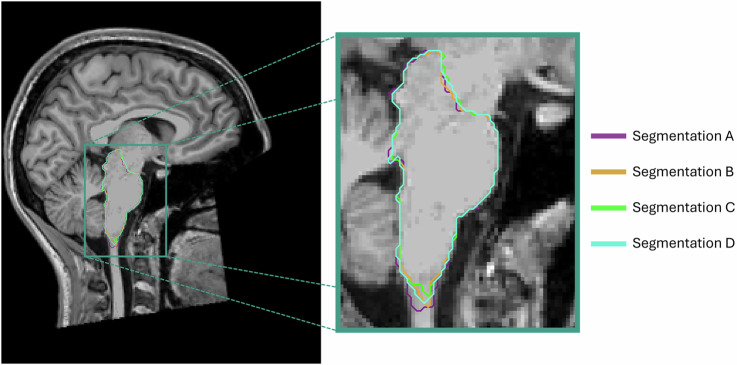
Example of segmentation variability. Different segmentations of the brainstem over a T1-weighted magnetic resonance image. Four different segmentation methods were used for generating these masks, creating slightly different versions of the output

No matter what method to use, segmentation variability must always be considered and quantified, since failing to do so can lead to inconsistencies in feature extraction. As observed in Fig. 3, different segmentation methods lead to slightly different segmentation outputs, which will directly affect the result of the posterior analysis. To objectively evaluate this variability, quantitative metrics are essential, as they provide standardized ways to assess consistency across segmentation methods and raters.

Among the most widely used metrics, the Dice similarity coefficient is highlighted, which quantifies the spatial overlap between segmentations, making it particularly useful for comparing manual annotations or evaluating DL-based methods [27]. The intraclass correlation coefficient, which evaluates agreement in continuous variables derived from segmented regions [27], or the Hausdorff distance, measuring the maximum boundary deviation between segmentations [28], may also be considered for this task. Additional metrics such as the Jaccard index, average surface distance, or mean surface distance can further improve the evaluation by capturing different aspects of segmentation accuracy.

In many scenarios, segmentation robustness can be further enhanced by employing multiple human raters or algorithms. When several experts or models independently segment the same images, their outputs can be harmonized using label fusion strategies. Simple approaches like majority voting assign each voxel to the most frequently chosen label, while more sophisticated methods, such as simultaneous truth and performance level estimation (STAPLE), estimate both the true segmentation and the reliability of each contributor [29]. Probabilistic fusion techniques, which incorporate uncertainty and confidence scores, offer even greater nuance, especially in complex or ambiguous regions. These strategies help mitigate individual biases and produce consensus segmentations that are more robust, reproducible, and suitable for downstream radiomic analysis.

## Step 5: Extract quantitative imaging features

**Fig. 4:**
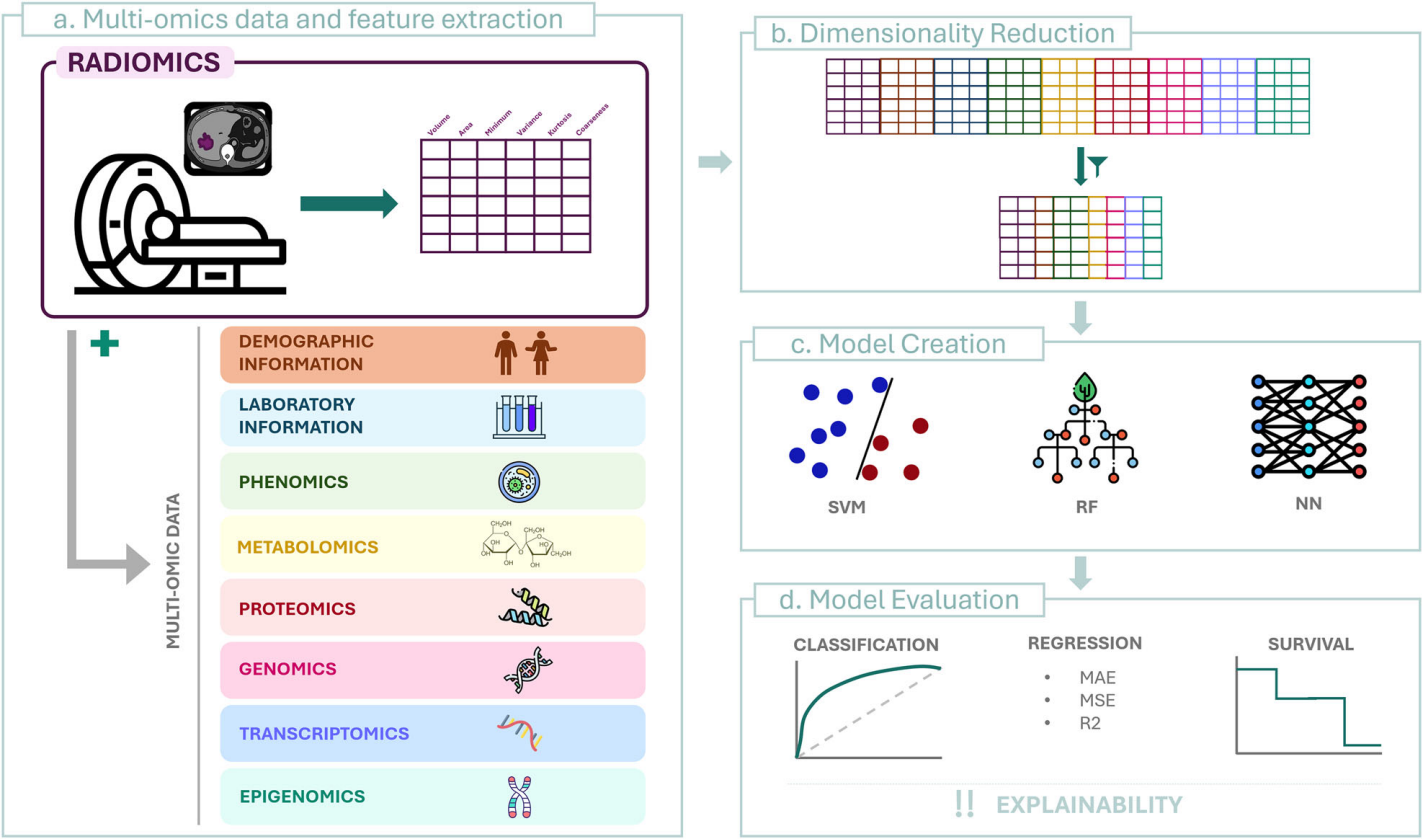
Radiomics and multi-omics integration for model creation. **a** This image shows how radiomics features from imaging data are combined with multi-omic information to create a complete dataset. **b** The dimensionality reduction process, which simplifies the high-dimensional data, retaining the most relevant features. **c** Some of the most used machine learning models. **d** The model’s evaluation for different tasks, with a focus on explainability for clinical integration

At this point, all the necessary elements have been collected and prepared for the heart of our study: the radiomic feature extraction (Fig. 4a). Images will be transformed into high-dimensional data. Precisely, each voxel that belongs to the ROI under study is evaluated mathematically to extract numerous quantitative descriptors, usually classified into two big groups: semantic and agnostic characteristics [2].

Semantic features are those that clinicians can intuitively understand and even use in their daily clinical routine, aligning with traditional radiology. These include, mostly, size and shape characteristics. On the other hand, agnostic features extract information that is not immediately apparent to the naked eye. These include texture features, which analyze the variations in pixel intensity, and wavelet features, which capture information across different scales and orientations of the image, among others. While agnostic features may lack direct clinical interpretation, they often reveal valuable markers that are not traditionally considered [2].

The number of features to extract is infinite. This, although it can be considered a strength, is a challenge, since variability across research studies is infinite. Different research studies may choose to focus on different sets of features, or even when the same features are used, they may be defined differently. This variability can lead to inconsistencies, making it difficult to compare results across studies or validate findings in different settings.

To address this issue, the Imaging Biomarker Standardization Initiative (IBSI) was established. The IBSI provides guidelines and standards for the extraction and reporting of high-throughput quantitative image analysis (*i.e*., radiomics), aiming to improve reproducibility and validation across studies [30]. This international initiative seeks to provide a uniform nomenclature and definitions for features, grouped into different groups, summarized in Table 1.

**Table 1:** Feature groups adapted from IBSI [30]

Feature family	Meaning	Count	Feature example
Morphology	Describes the geometry (shape and structure) of the ROI.	29	Volume, surface area, sphericity
Local intensity	Captures the intensity of pixels in a neighborhood of voxels.	2	Local and global intensity peak
Intensity-based statistics	Distribution of the voxels’ intensity within the ROI.	18	Mean or median intensity, intensity kurtosis
Intensity histogram	Distribution of the discretized intensity distribution.	23	Discretized intensity variance, maximum discretized intensity
Intensity-volume histogram	Intensity distribution relative to volume.	5	Intensity at volume fraction
Gray-level co-occurrence matrix	Measures texture by considering the spatial relationship of pixels.	25	Sum average, contrast, cluster shade
Gray-level run length matrix	Measures texture by analyzing consecutive pixels with the same intensity.	16	Long run emphasis, gray-level non-uniformity
Gray-level size zone matrix	Measures texture by examining the size of zones with the same intensity.	16	Large zone emphasis, zone percentage
Gray-level distance zone matrix	Measures texture by examining the size of zones with the same intensity and distance to the ROI limit.	16	Small distance emphasis, gray-level variance
Neighborhood gray tone difference matrix	Measures texture by calculating the difference between neighboring pixels.	5	Coarseness, complexity
Neighboring gray-level dependence matrix	Measures texture by analyzing the roughness between neighboring pixel intensities.	17	Dependence count variance, dependence count energy

*ROI* Region of interest

These features may not only be derived from the original image, but also from filtered images. Applying various image filters, such as wavelet transforms, Laplacian of Gaussian, or others, can enhance specific image features or reveal additional patterns that are not visible in the original images, providing better predictive power [31].

Careful attention must be paid to the hyperparameters used during feature extraction, as they can significantly influence the reproducibility and interpretability of the results. Key parameters include image-type-specific parameters, like the sigma value in Gaussian-based filters, or kernel sizes for texture feature definitions. It is advisable to document all hyperparameter choices explicitly and, when possible, perform sensitivity analyses to assess their impact on feature stability.

There are several software tools available for performing radiomic feature extraction, with PyRadiomics being one of the most widely used. PyRadiomics is an open-source Python package that allows the extraction of a wide range of radiomic features from medical images following IBSI standards and providing flexibility in feature selection and customization [32]. However, PyRadiomics is not the only tool available. Other engines, such as MATLAB, also offer radiomic feature extraction capabilities [33]. The choice of software may depend on the specific requirements of the study, the available resources, and the level of customization needed. Regardless of the software used, it is essential to ensure that the extraction process is consistent and adheres to standard protocols.

In addition to the IBSI guidelines, several complementary frameworks have been developed to strengthen the reproducibility and quality of radiomic studies. One such initiative is the Quantitative Imaging Biomarkers Alliance‒QIBA [34], which focuses on ensuring the clinical reproducibility of quantitative biomarkers. Two widely recognized scoring systems are the Radiomics Quality Score‒RQS [35], its updated version RQS2 [36], and METhodological RadiomICs Score‒METRICS [37]. These frameworks help assess the methodological rigor of radiomic research by encouraging best practices. They serve as valuable tools for identifying potential weaknesses and guiding improvements in study design and reporting. Alongside these scoring systems, the CheckList for EvaluAtion of Radiomics research‒CLEAR [38, 39] provides a structured set of reporting guidelines to ensure completeness in radiomics publications. By outlining key elements that should be transparently documented, CLEAR facilitates critical appraisal and comparability across studies. Together, these initiatives complement IBSI by promoting not only standardized feature extraction but also high-quality and reproducible radiomic research.

## Step 6: Prioritize feature selection and dimension reduction

Feature extraction often yields a large number of features, many of which may lack significant clinical relevance. Following standard procedures, such as those outlined by IBSI guidelines, up to 172 features are extracted only from the original image, a number that can be significantly augmented by using filtered versions of the original images.

When working with this large number of features, one of the primary concerns is the curse of dimensionality, which occurs when the number of features (dimensions) exceeds the number of observations (samples). This can lead to challenges in analysis and interpretation, especially when working with small datasets and predictive models, where overfitting becomes a concern.

A common guideline to avoid this issue is the so-called Rule of 10N, which suggests that the number of features used in a model should not exceed 10% of the number of observations in the class with the fewest elements. For example, if you have 70 patients, 40 in one class and 30 in the other, the number of features should not exceed 3. The bigger isn’t always the better; having more features can complicate the analysis without necessarily improving results, therefore strategies in feature selection and dimensionality reduction must be considered (Fig. 4b).

Feature selection methods are designed to identify the most relevant features from the original set, often using mathematical criteria such as correlation coefficients between characteristics to delete the most related ones, or importance metrics that consider the relationship between the characteristics and the target, like maximum relevance minimum redundancy algorithms or mutual information-based techniques [40]. This way, only features containing significant information will be retained, filtering out noise and irrelevant or redundant information. This not only simplifies posterior models but also improves their interpretability, making it easier to draw meaningful conclusions.

Another approach is feature combination, where the original feature space is transformed to produce a new, smaller set of relevant features. Techniques like principal component analysis (PCA) and linear discriminant analysis (LDA) are commonly used for this purpose. The former, for instance, reduces dimensionality by identifying directions (principal components) in which the data varies the most, allowing the retention of the most important aspects of the data while discarding the less informative ones [41]. The latter, on the other hand, seeks to maximize the separation between different classes, making it particularly useful when the goal is to distinguish between different clinical outcomes [42].

The choice between feature selection and feature combination, or a combination of both, depends on the specific context of the study and the nature of the data [43]. Feature selection is often preferred when interpretability is crucial, as it retains the original features that are most predictive. Among this group, methods that do not consider the output may be preferred, as they provide a more unbiased view of the relationships among features. On the other hand, feature combination, while potentially more powerful in reducing dimensionality, can lead to new features that are less interpretable but capture the essential variability in the data.

Feature selection and combination algorithms are available in programming languages like R or Python, particularly through libraries such as scikit-learn [19].

## Step 7: Consider integration of clinical and multi-omics data

When performing radiomic studies, the integration of additional data types, such as clinical and demographic information, as well as more advanced datasets like genomics or proteomics, is becoming essential (Fig. 4a). Data in any form (whether it is patient history, age, gender, genetic profiles, or protein expression levels) can significantly enhance the relevance of our analyses [44].

The benefits of integrating such data are numerous. A key advantage is the potential for improved predictive models since models receive a wider view of a patient’s condition, achieving more accurate and comprehensive results [44]. Additionally, the inclusion of clinical, demographic, and biological data can facilitate the discovery of new biomarkers that link imaging features with biological processes that might remain hidden if radiomic features were analyzed in isolation. For example, the combination of imaging markers with genomic data might reveal associations between specific genetic mutations and certain radiomic traits, enhancing the value of medical imaging for the diagnosis and treatment of patients.

However, the inclusion of additional data types in radiomic studies comes with some challenges. One of the primary concerns is the potential for data overload, where the volume and complexity of the information can complicate analysis and interpretation. Moreover, the integration of different data types, particularly those with varying scales and formats, can introduce noise and biases if not handled carefully. For example, combining imaging features with raw clinical data or genomic profiles without harmonization may lead to spurious correlations or model instability.

To mitigate these risks, it is essential that the included data is not used in its raw form but is instead preprocessed using standard data analysis techniques. This includes one-hot encoding for categorical variables, z-score normalization or min-max scaling for continuous variables, and imputation strategies for missing values.

A common pitfall is the assumption that all data types contribute equally to model performance. In reality, some variables may dominate due to scale or redundancy, leading to biased predictions. Therefore, feature selection methods should be applied to identify the most informative variables across modalities.

## Step 8: Construct predictive models with machine learning techniques

At this point, a ‘clean’ dataset with meaningful radiomic variables, optionally combined with other data, is constructed. These features, often too complex to be interpreted by the human eye, hold the potential to reveal disease characteristics and assess treatment response and patient outcomes, among others. To exploit this potential, predictive modeling through AI plays a crucial role in analyzing these data. Although this is not a guide on how to build AI models, the key concepts, most used methods, and best practices will be outlined to properly perform a radiomics-based study.

In radiomics, several types of machine learning (ML) models are commonly used for predictive tasks, including, but not limited to, random forest, support vector machine, and logistic regression (Fig. 4c) [45]. These models are widely selected because of their robustness and ability to handle high-dimensional data while maintaining certain simplicity. Support vector machine, for instance, is particularly effective in high-dimensional spaces, making it ideal for radiomic data; random forest, with its ensemble approach, is useful for capturing the importance of different features and handling overfitting [46, 47]. Logistic regression, while simpler, remains a powerful tool for binary classification tasks often encountered in radiomic studies. Nonetheless, other models, including gradient boosting or multilayer perceptron, can also be employed [45]. Radiomics is not immune to the rise of DL methods. As DL models advance, they are increasingly being integrated into radiomic workflows under the term of deep radiomics [48]. In this sense, there are two primary approaches: the first involves using DL models on pre-filtered radiomic features, where these features serve as inputs to the neural network. This method uses the existing radiomic framework while benefiting from the predictive power of deep learning. The second approach is more direct, simplifying the presented workflow, where DL models are applied directly to the imaging data using convolutional neural networks, reducing the feature extraction steps.

Each algorithm has its own list of advantages and disadvantages, and sometimes, it is difficult to select the most appropriate one for our particular task. However, in this case, we do not need to choose; instead, several may be used. This is what is called an ensemble model, which combines the predictions from multiple models to improve overall performance since it benefits from the diverse perspectives provided by different models [49].

The selection of the strategy to follow when constructing the model relies on the individual researcher and their expertise. However, the following good practices should be considered to guarantee their quality.

### K-fold cross-validation, leave-one-out cross-validation, stratified and repeated stratified cross-validation

K-fold cross-validation involves dividing the dataset into K subsets and using each subset as a validation set while training the model on the remaining data. This method helps in evaluating the model’s stability and performance across different data splits. In stratified K-fold cross-validation, the division of the data preserves the class distribution within each fold, ensuring a balanced representation of each class in both training and validation sets. Repeated stratified cross-validation repeats this process multiple times to account for variability in the data split and provides more robust performance estimates. Leave-one-out cross-validation, on the other hand, is a more exhaustive method where the model is trained on all but one sample, which is then used for validation [50].

### Hyperparameter tuning

It involves searching for the best set of parameters that optimizes model performance, should be applied. This can be achieved through grid search, random search, or more sophisticated methods like Bayesian optimization [51].

### Careful handling of class imbalance

A common practice in radiomic datasets, especially when dealing with medical conditions where certain outcomes are rare. This can be done when constructing the dataset or using oversampling, *e.g*., using the synthetic minority oversampling technique (SMOTE), or downsampling (preferred) algorithms [52].

All these considerations are collected in different Python libraries, such as scikit-learn, which offers comprehensive tools for ML model development, validation, and hyperparameter tuning. For DL applications, TensorFlow and PyTorch are powerful frameworks that support both the development of complex neural networks and the integration of DL into radiomic studies. Nonetheless, other programming languages like MATLAB or C++ might be useful in this step.

## Step 9: Evaluate model performance using appropriate metrics

When evaluating the models’ performance in radiomic studies, it is essential to choose metrics that align with the specific nature of the task (Fig. 4d). In classification problems, where the goal is to categorize data into distinct groups (*e.g*., distinguish between benign and malign masses), metrics such as accuracy, precision, recall, F1 score, positive predictive value, negative predictive value, and receiver operating characteristic‒ROC analysis are often employed. These metrics usually reflect the model’s ability to correctly identify true positives and true negatives. The choice of metric should also reflect the clinical priorities of the task. For instance, in cancer screening scenarios, minimizing false negatives (*i.e*., cases where malignant lesions are incorrectly classified as benign) is crucial, as these errors can delay diagnosis and treatment. In such cases, recall (*i.e*., sensitivity) becomes a key metric, as it measures the proportion of actual positives correctly identified by the model. On the other hand, in pre-surgical planning, where a false positive might lead to unnecessary invasive procedures, precision is more relevant, as it quantifies the proportion of positive predictions that are truly positive.

However, beyond discrimination, it is equally important to assess calibration, which evaluates how well predicted probabilities reflect actual outcomes [53]. Calibration curves and the Brier score are commonly used for this purpose. Calibration curves visually compare predicted probabilities with the frequency of observed outcomes, while the Brier score quantifies the accuracy of probabilistic predictions by measuring the mean squared difference between predicted probabilities and actual outcomes [54]. Together, these metrics provide a more comprehensive understanding of model reliability in clinical settings.

Regression problems, which aim to predict continuous outcomes (*e.g*., approximate the value of a serum marker based on radiomic features), require metrics like mean squared error (MSE), mean absolute error (MAE), and R-squared. These metrics evaluate how closely the predicted values are to the real values. For survival analysis, where the focus is on time-to-event data, metrics such as the concordance index (C-index) are pivotal. These metrics assess the model’s ability to correctly rank survival times and evaluate the calibration of predicted survival probabilities over time [55, 56].

While these quantitative metrics are important, they represent just one aspect of the model evaluation process in radiomic studies. Only focusing on these numbers can be misleading if other critical factors are overlooked.

Besides standard metrics, the explainability of the model is a critical consideration in clinical practice, in general, and in radiomic studies, in particular. Understanding how models arrive at their predictions can be as important as obtaining good-quality metrics. Feature importance analysis techniques such as SHapley Additive exPlanations (SHAP) [57] or Local Interpretable Model-agnostic Explanations (LIME) [58] allow us to understand the model’s decision-making process, revealing the most meaningful characteristics of our dataset to study their clinical relevance. Feature importance analysis, found in ML models like random forest, highlights the most influential features that contribute to the model’s predictions. On the other hand, SHapley Additive exPlanations values, which are based on cooperative game theory and can be computed on every AI model, offer a more nuanced interpretation by quantifying the contribution of each feature to each prediction, ensuring transparency in model interpretation [58]. These techniques, and other eXplainable AI (XAI) methods, which aim to explain the inner workings of DL black boxes [59], can be additionally applied to deep radiomics studies. Another fundamental aspect of evaluating model performance in radiomics relies on its robustness (*i.e*., the ability of generalization on unseen data). This is particularly important in radiomics, where variations in imaging protocols, scanner types, and patient populations can introduce significant variability, leading to incorrect predictions. Tensor radiomics represents an emerging approach to enhance the robustness of radiomic models. By considering variations on the feature extraction protocol (*i.e*., modifying the extraction parameters), multiple values for the same feature are obtained, leading to models that are less sensitive to variations and more capable of generalizing across different settings [60].

This multidimensional evaluation is essential for translating radiomic research into meaningful clinical conclusions and developing models that, besides being accurate, are trustworthy and applicable in daily clinical practice.

## Step 10: Translate models into clinical practice and workflow integration

Although most radiomic studies excel through the development and evaluation stages, they often forget their ultimate goal: integrating these models into clinical practice. The primary purpose of radiomic research is not merely to create accurate models and publish a scientific paper, but to implement them in real-world settings to improve patient care. Unfortunately, many studies stop at evaluation, proposing promising tools that never reach the real patient.

This is not without a reason: the incorporation of radiomic tools in a real clinical setting comes with numerous challenges, including exhaustive clinical validation, compliance with health information systems standards, and ethical considerations, among others [61, 62]. Moreover, these algorithms are classified as medical devices and must adhere to the Medical Devices Regulation [63] to ensure they can be safely and effectively used in clinical practice.

One key factor that is often overlooked is the clinical workflow itself. Radiomic models should be embedded into the clinician’s existing workstation with minimal disruption. This requires ensuring that the tool integrates completely with existing software and does not introduce additional complexity or slow down decision-making processes. To achieve this, modern containerization technologies such as Docker or Kubernetes can be used. Docker allows radiomic tools to be packaged with all their dependencies into containers, ensuring that they run across different environments [64]. Kubernetes further enhances this by orchestrating the deployment and management of these containers across various nodes, ensuring that resources are optimally used, and the system can work even under high workloads [64].

Moreover, the interface must be intuitive and user-friendly, providing clear and actionable information. Real-time analysis or fast processing is critical to maintain the pace of clinical operations. The focus should be on enhancing clinical decision-making rather than adding new layers of data that could overwhelm the physician. Automated report generation and standardization of outputs can help in this regard.

Open science practices, including the developed software and code together with trained models, also play a crucial role in facilitating clinical integration [65]. By promoting transparency, reproducibility, and collaboration, these practices can accelerate validation efforts, improve trust and reduce barriers to adoption. Publicly available resources allow institutions to adapt models to their specific workflows, ultimately supporting broader implementation.

While progress has been made [66], there are still significant challenges to fully translating radiomic models into routine clinical practice. This field needs to be further explored, with a focus on optimizing integration, usability, and scalability to ensure that these tools not only meet the standards of healthcare systems but also enhance patient outcomes.

## Conclusions

Radiomics holds the potential to revolutionize clinical practice by transforming medical images into quantifiable data. Its capacity to uncover hidden patterns and correlations can significantly enhance the diagnosis, prognosis, and treatment of diseases, pushing the boundaries of personalized medicine. However, for radiomics to reach its potential, overcoming challenges related to standardization, reproducibility, and clinical integration is essential. In this work, we presented a practical recipe that serves as a guide on how to perform radiomic studies, settling the basis for the standardization of the field.

## Data Availability

This work does not include original data. No datasets were generated or analyzed.

## Abbreviations

AI: Artificial intelligence
CT: Computed tomography
DL: Deep learning
IBSI: Imaging Biomarker Standardization Initiative
ML: Machine learning
MRI: Magnetic resonance imaging
PET: Positron emission tomography
ROI: Region of interest
